# Transcription factor TCF3 promotes bladder cancer development via TMBIM6-Ca^2+^-dependent ferroptosis

**DOI:** 10.1038/s41420-025-02585-8

**Published:** 2025-07-03

**Authors:** Wei-Feng Yang, Wei-Ming Guo, Qing-Tian Luo, Jingfen Lu, Zhou-Ke Tan, Yuan-Chun Ye, Gang Fan

**Affiliations:** 1 Department of Urology, Qianhai Shekou Free Trade Zone Hospital, Shenzhen, China; 2 Affiliated Nanshan Hospital of Shenzhen University, Shenzhen, Guangdong China; 3 Department of Sport Medicine, Affiliated Nanshan Hospital of Shenzhen University, Shenzhen, Guangdong China; 4 Department of Gastroenterology and Endoscopy Center, Affiliated Nanshan Hospital of Shenzhen University, Shenzhen, Guangdong China; 5 The First Clinical Medical College, Guangzhou University of Chinese Medicine, Guangzhou, Guangdong China; 6 Medical Research Center, Affiliated Nanshan Hospital of Shenzhen University, Shenzhen, Guangdong China; 7 School of Science, Shenzhen Campus of Sun Yat-Sen University, Shenzhen, China

**Keywords:** Cancer models, Cell biology, Bladder cancer

## Abstract

TMBIM6, a Ca^2+^ channel-like protein, shows an increased expression in numerous types of cancer. However, no study has reported its role in bladder cancer. This study aimed to explore the roles and mechanisms of TMBIM6 in bladder cancer. TMBIM6, ferroptosis-related proteins (GPX4, SLC7A11, and FTH1), and calmodulin (CaM) expressions in bladder cancer and paracancerous tissues were obtained by immunohistochemistry. The bladder cells were overexpressed or silenced with TCF3/TMBIM6 with ferroptosis inducer (Erastin)/Ca^2+^ blocker (BAPTA-AM) to investigate the effects on Ca^2+^-dependent ferroptosis and other functions. Finally, tumorigenicity was validated in nude mice. TMBIM6 and ferroptosis-related proteins were up-regulated in bladder cancer tissues, but CaM was downregulated. TMBIM6 overexpression enhanced proliferation, invasion, migration, GSH/GPX4 levels, and ferroptosis resistance while suppressing MDA, Fe²⁺, and lipid ROS in bladder cancer cells, effects reversed by Erastin. TCF3 was up-regulated in cancer and enriched in Ca^2+^ and ferroptosis-related pathways. TCF3 directly interacted with TMBIM6 and transcriptionally activated TMBIM6 expression. Both TCF3 and TMBIM6 overexpression exhibited comparable effects in modulating ferroptosis and other cellular processes, whereas TMBIM6 knockdown effectively reversed these phenotypic alterations. In addition, silencing TCF3 upregulated Ca^2+^ and CAM levels, while BAPTA-AM reversed these changes. In vivo, ov-TCF3 promoted tumor volume, weight, and TMBIM6 expression, and inhibited Ca^2+^ concentration, while Erastin reversed these changes. Our findings demonstrate that TCF3 facilitates bladder cancer progression through the enhancement of TMBIM6-Ca^2+^-mediated ferroptosis resistance. Both TCF3 and TMBIM6 emerge as promising biomarkers and therapeutic targets for bladder cancer intervention.

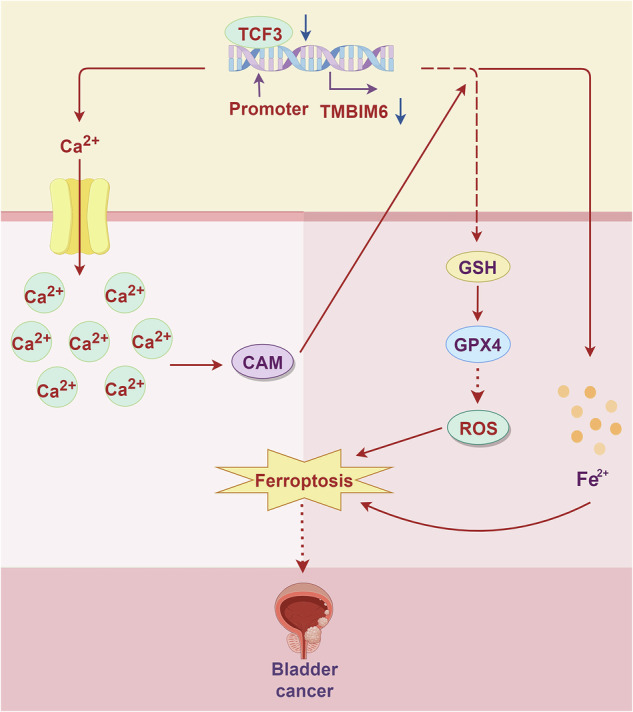

## Introduction

Bladder cancer, a genitourinary malignancy with a high morbidity and mortality rates, exhibits a higher incidence in male patients compared to females [1, 2]. The pathogenesis of bladder cancer involves complex mechanisms, including metabolic disorders, genetic alterations, inflammatory responses, oxidative stress, and immune dysregulation [3–5]. Traditional cancer treatments, including chemotherapy and hormone therapy, may induce adverse side effects in patients, such as retinal damage [6]. Over the last decade, advances in sequencing technologies have significantly enhanced our understanding of bladder cancer biology. These developments have led to the emergence of novel therapeutic strategies beyond traditional surgery and radiotherapy, particularly immune checkpoint inhibitors and molecularly targeted therapies [7]. While these technological advancements offer new hope for cancer treatment, overcoming cancer remains one of the most significant challenges in modern medicine. Therefore, the identification of novel biomarkers and therapeutic targets continues to be crucial for advancing bladder cancer treatment.

Ferroptosis, a newly discovered form of iron-dependent cell death, is characterized by impaired intracellular lipid oxide metabolism and ion-catalyzed metabolic abnormalities, leading to the accumulation of lipid reactive oxygen species (ROS), which subsequently causes intracellular redox imbalance and ultimately induces cell death. This process has been found to be closely associated with the development and therapeutic response of various tumors [8]. A range of therapeutic agents, including experimental compounds, approved pharmaceuticals, ionizing radiation, and cytokines, such as Erastin, sorafenib, statins, and artemisinin, can inhibit cancer cell proliferation through the induction of ferroptosis [9, 10]. In addition, some natural compounds, such as baicalin and erianin, can inhibit bladder cancer development by inducing ferroptosis [11, 12]. In conclusion, targeting ferroptosis to impede bladder cancer progression represents a promising and innovative therapeutic strategy in oncology research.

Intracellular calcium ions (Ca^2+^), serving as crucial second messengers, play a pivotal role in regulating diverse cellular cell functions [13–15]. Disruptions in intracellular Ca^2+^ homeostasis have been extensively linked to various oncogenic processes, including tumorigenesis, angiogenesis, tumor development, and metastasis [13]. Notably, abnormal Ca^2+^ concentration is an important risk factor for the development of bladder cancer. Interestingly, elevated intracellular Ca^2+^ concentration promotes apoptosis and inhibits the development of bladder cancer [16, 17]. Mechanistically, enhanced increased Ca^2+^ influx induces matrix metalloproteinase-mediated hyperpolarization, which subsequently accelerates lipid peroxidation, thus causing cellular ferroptosis known as Ca^2+^-dependent ferroptosis [18]. Despite these advances, the specific role and mechanisms of Ca^2+^-dependent ferroptosis in bladder cancer have not been reported yet.

Transmembrane B-cell lymphoma 2-associated X protein (Bax) inhibitor motif-containing 6 (TMBIM6), alternatively referred to as Bax inhibitor-1, represents a highly conserved transmembrane protein predominantly localized to the endoplasmic reticulum (ER) membrane [19, 20]. Functioning as a Ca^2+^ channel-like protein, TMBIM6 has been demonstrated to exhibit upregulated expression profiles across multiple cancer types, particularly in breast cancer, glioblastoma, and non-small cell lung cancer [21, 22]. Notably, Kim et al. provided compelling evidence that pharmacological modulation of TMBIM6-mediated Ca^2+^ leakage effectively suppresses both tumorigenesis and cancer progression, thereby establishing a novel therapeutic paradigm for malignant tumor treatment [22]. Despite these significant advances, the functional significance and molecular mechanisms of TMBIM6 in bladder cancer pathogenesis remain unexplored. In this study, we systematically investigated the expression profile of TMBIM6 in bladder cancer and further elucidated its regulatory role in Ca^2+^-dependent ferroptosis pathways.

## Results

### Differential expression analysis of TMBIM6, CAM, and ferroptosis-related proteins in bladder cancer tissues

**Fig. 1:**
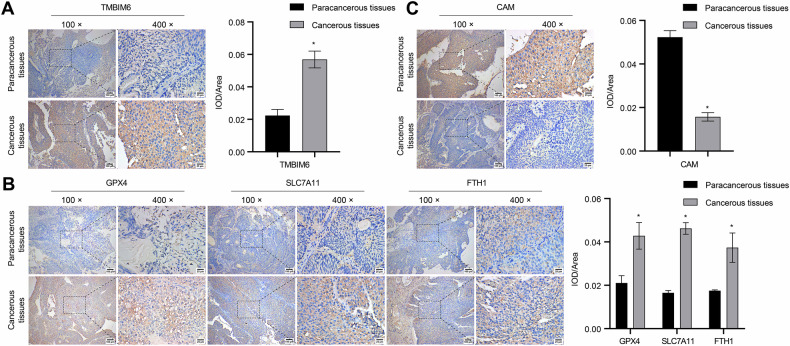
The expressions of TMBIM6, CAM, and ferroptosis-related proteins in bladder cancer tissues. **A**–**C** The expressions of TMBIM6, CAM, and ferroptosis-related proteins were analyzed by IHC. *p < 0.05, vs. Paracancerous tissue.

TMBIM6 is known to mediate the Ca^2+^ pathway regulating the progression of multiple cancers, including cervical, breast, lung, and prostate cancers [22]. Ca^2+^-dependent ferroptosis regulates lung cancer cell proliferation and migration functions [23]. Based on the above background, the expressions of TMBIM6, ferroptosis-related proteins (GPX4, SLC7A11, FTH1), and CaM in paracancerous and cancerous tissues were analyzed by IHC. The expression of TMBIM6 and ferroptosis-related proteins was significantly higher in cancerous tissues, while CAM was significantly decreased (Fig. 1A–C).

### si-TMBIM6 inhibits the proliferation and migration of bladder cancer cells by inducing ferroptosis

**Fig. 2:**
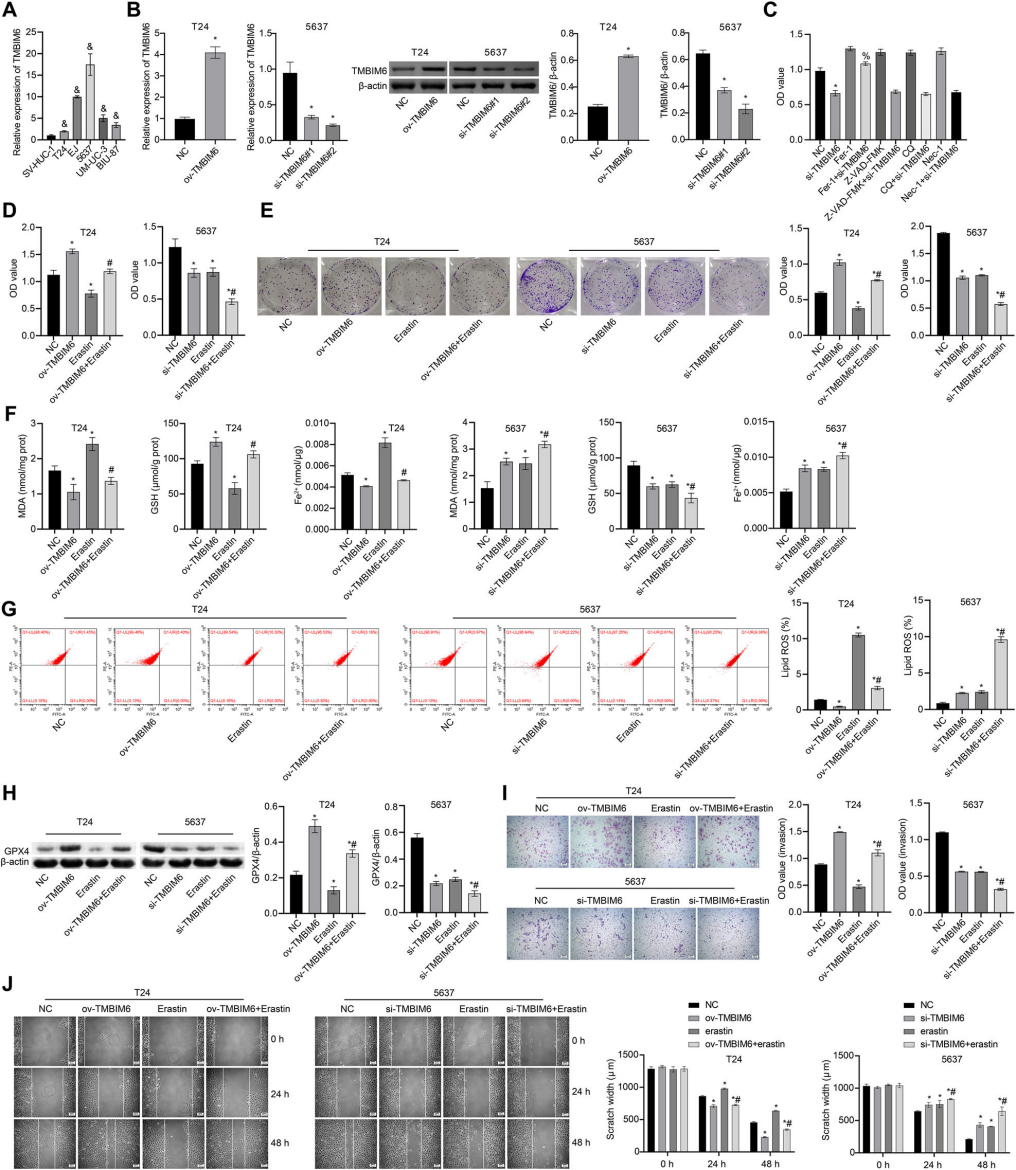
si-TMBIM6 inhibits the bladder cancer cells’ progress by inducing ferroptosis. **A**, **B** The TMBIM6 levels were assayed by qRT-PCR and western blot. **C** The effects of ferroptosis, apoptosis, autophagy, and necroptosis inhibitors on the proliferative capacity of cells in the si-TMBIM6 group were obtained by CCK8. **D**, **E** The proliferative capacity was detected by CCK8 and colony formation assays, respectively. **F** The MDA, GSH, and Fe^2+^ levels were measured using biochemical assay kits. **G** Lipid ROS levels were detected by a C11-BODIPY fluorescent probe. **H** GPX4 expression was obtained by western blot. **I**, **J** Invasion and migration abilities were detected and quantified by Transwell and scratch assays, respectively (scale bar: 100 μm). *p < 0.05, vs. NC; ^#^*p* < 0.05, vs. Erastin.

Next, we examined the expression of TMBIM6 in SV-HUC-1 and bladder cancer cell lines (T24, EJ, 5637, UM-UC-3, BIU-87). Initially, we evaluated the expression of TMBIM6 in SV-HUC-1 and various bladder cancer cell lines (T24, EJ, 5637, UM-UC-3, BIU-87). Our findings revealed elevated TMBIM6 expression in bladder cancer cell lines compared to the SV-HUC-1 group, with 5637 cells exhibiting the highest levels and T24 cells showing the lowest (Fig. 2A). Consequently, T24 and 5637 cells were chosen for further experiments. Subsequently, TMBIM6 was either overexpressed or inhibited in T24 and 5637 cells to validate its effects on bladder cancer cells. The CCK8 assay results demonstrated that si-TMBIM6 reduced cell proliferation, and notably, only Fer-1 managed to rescue the proliferative capacity of the si-TMBIM6 group (Fig. 2B). This led us to speculate that si-TMBIM6 may influence the proliferation of bladder cancer cells by promoting ferroptosis. To delve deeper into the role of TMBIM6 in ferroptosis within bladder cancer cells, we conducted additional experiments. In T24 cells, overexpressing TMBIM6 increased cell proliferation and GSH levels, while decreasing MDA and Fe^2+^ levels; however, these effects were counteracted by Erastin. Conversely, in 5637 cells, si-TMBIM6 and Erastin suppressed cell proliferation and GSH levels, and elevated MDA and Fe^2+^ levels. Notably, the combination of si-TMBIM6 and Erastin exhibited the most pronounced effects (Fig. 2C–F). Furthermore, in T24 cells, the ov-TMBIM6 group displayed reduced lipid ROS levels, scratch width, and increased GPX4 expression and invasion capabilities as compared to the NC group; nonetheless, these effects were reversed by Erastin. Conversely, in 5637 cells, lipid ROS and scratch width were significantly increased in the si-TMBIM6 and Erastin groups, leading to reduced GPX4 expression and invasion ability. The combined treatment demonstrated the most potent effects (Fig. 2G–J). Collectively, our findings suggest that si-TMBIM6 impedes bladder cancer cell proliferation and migration by inducing cellular ferroptosis.

### TCF3 orchestrates TMBIM6 expression through transcriptional regulation

**Fig. 3:**
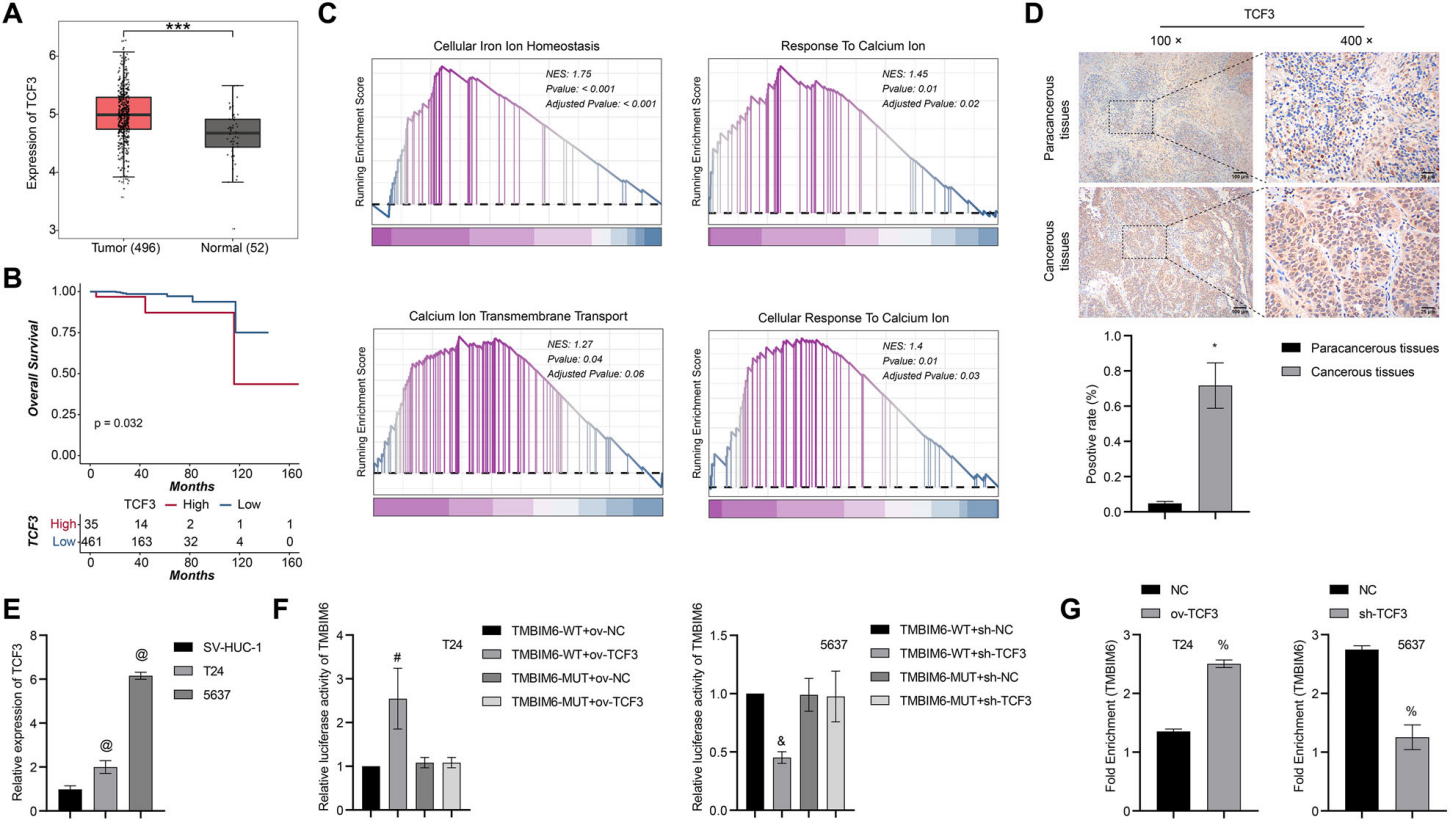
TCF3 functions as a transcriptional regulator of TMBIM6 expression. **A** The expression of TCF3 in the TCGA database; **B** Survival analysis of TCF3; **C** GSEA analysis; **D**, **E** TCF3 expression was measured by IHC and qRT-PCR; **F**, **G** The interaction of TCF3 with TMBIM6 was verified by dual-luciferase reporter and ChIP assays; ***p < 0.05, vs. Normal; *p < 0.05, vs. paracancerous tissues; ^@^*p* < 0.05, vs. SV-HUC^-^1; ^#^*p* < 0.05, vs. TMBIM6-WT + oe-NC; ^&^*p* < 0.05, vs. TMBIM6-WT + sh-NC; ^%^*p* < 0.05, vs. NC.

TCF3, a transcription factor, has been reported to be upregulated in prostate cancer and positively associated with cancer cell development [24]. To investigate the potential variation of TCF3 expression in bladder cancer, we analyzed TCF3 expression in both control and cancerous tissues within the TCGA database. Our findings revealed high expression of TCF3 in cancer tissues, with a shorter overall survival observed in the high-expression TCF3 group compared to the low-expression group (Fig. 3A, B). TCF3 appeared to be predominantly involved in cellular iron ion homeostasis, response to calcium ions, calcium ion transmembrane transport, and cellular response to calcium ion pathways (Fig. 3C). Notably, TCF3 expression was significantly elevated in cancerous tissues (Fig. 3D). Moreover, the expression of TCF3 was notably increased in T24 and 5637 cells (Fig. 3E). Subsequent ChIP and dual-luciferase reporter assays indicated that ov-TCF3 promoted the binding of TCF3 to the TMBIM6 promoter, whereas sh-TCF3 had the opposite effect, suggesting a direct involvement of TCF3 in the regulation of TMBIM6 expression (Fig. 3F, G). These results suggest that TCF3 may play a key role in modulating TMBIM6 expression.

### TCF3 affects bladder cancer cell function through ferroptosis

**Fig. 4:**
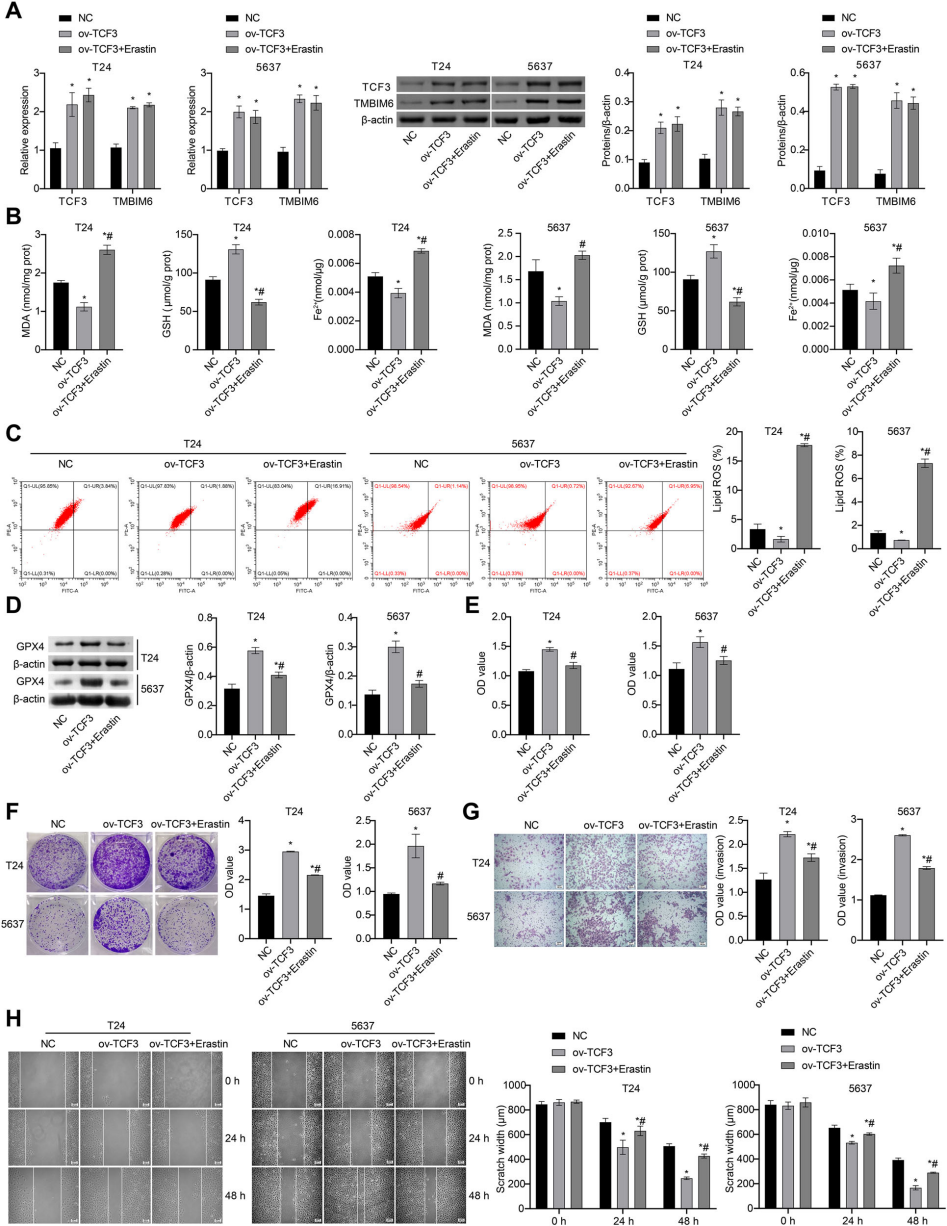
Erastin inhibits the effects of ov-TCF3 on bladder cancer cell function. **A** The TCF3 and TMBIM6 levels were measured by qRT-PCR and western blot. **B** MDA, GSH, and Fe^2+^ levels were measured using biochemical assay kits. **C** Lipid ROS levels were measured by C11-BODIPY fluorescent probe. **D** GPX4 expression was obtained by western blot. **E**, **F** The proliferation ability was examined by CCK8 and colony formation assays, respectively. **G**, **H** The invasion and migration abilities were detected and quantified by Transwell and scratch assays, respectively (scale bar: 100 μm). *p < 0.05, vs. NC; ^#^*p* < 0.05, vs. ov- TCF3.

To further investigate whether TCF3 modulates bladder cancer cell function through ferroptosis, we overexpressed TCF3 in bladder cancer cells and treated them with Erastin. Initially, T24 and 5637 cells were successfully overexpressed with TCF3. Subsequent analysis revealed a significant upregulation of TMBIM6 expression following TCF3 overexpression (Fig. 4A). Comparative analysis with the NC group demonstrated that TCF3-overexpressing cells exhibited significant reductions in MDA content, Fe²⁺ concentration, lipid ROS accumulation, and scratch wound closure rate. In contrast, these cells displayed markedly elevated GSH and GPX4 expression levels, along with enhanced proliferative and invasive capacities. Erastin could significantly reverse the above changes (Fig. 4B–H). The above results suggest that TCF3 may affect cell function through ferroptosis.

### TCF3 inhibits cellular ferroptosis through TMBIM6, consequently promoting cell development

**Fig. 5:**
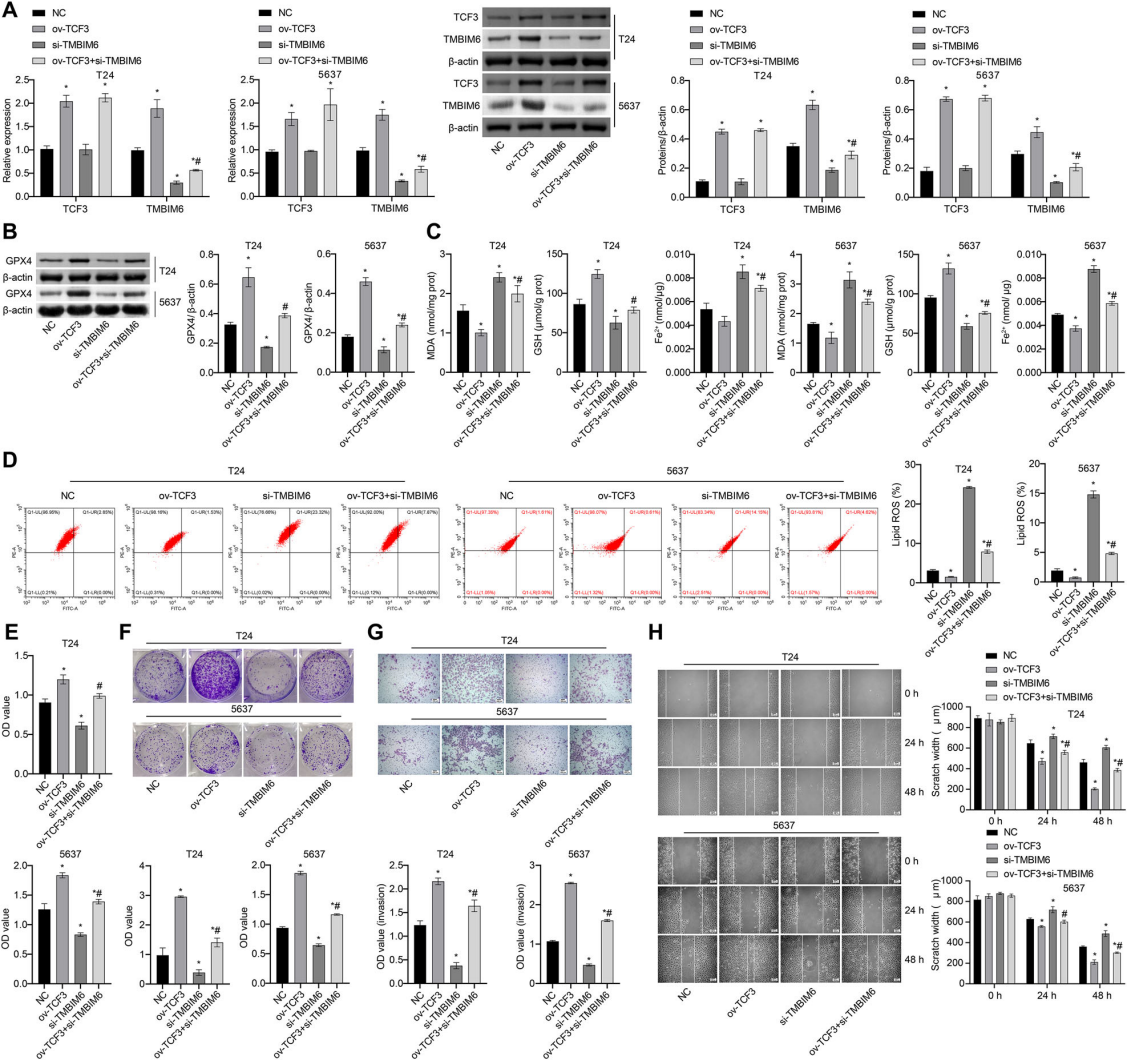
TCF3 inhibits cellular ferroptosis through TMBIM6, consequently promoting cell development. **A** TMBIM6 expression levels were measured by qRT-PCR and western blot. **B** GPX4 expression was obtained by western blot. **C** MDA, GSH, and Fe^2+^ levels were measured using biochemical assay kits. **D** Lipid ROS levels were measured by a c11-bodipy fluorescent probe. **E**, **F** The proliferation ability was measured by CCK8 and colony formation assays, respectively. **G**, **H** The invasion and migration abilities were measured and quantified by Transwell and scratch assays, respectively (scale bar: 100 μm). *p < 0.05, vs. NC; ^#^*p* < 0.05, vs. ov-TCF3.

To investigate whether TCF3 influences cellular ferroptosis through TMBIM6, we treated the cells with ov-TCF3, si-TMBIM6, and a combination of ov-TCF3+si-TMBIM6. Ov-TCF3 upregulated the expression of TMBIM6. The ov-TCF3+si-TMBIM6 group showed significantly lower TMBIM6 expression compared to the ov-TCF3 group (Fig. 5A). In comparison to the NC group, the ov-TCF3 group exhibited increased levels of GPX4 and GSH, as well as enhanced cell proliferation and migration abilities, while levels of MDA, Fe^2+^, and lipid ROS, as well as scratch width, were reduced. However, these changes were reversed with ov-TCF3+si-TMBIM6 treatment (Fig. 5B–H). These results suggest that TCF3 may potentially inhibit cell ferroptosis via TMBIM6, thereby promoting cell development.

### sh-TCF3 stimulates bladder cancer cell ferroptosis by inducing intracellular Ca^2+^ concentration

**Fig. 6:**
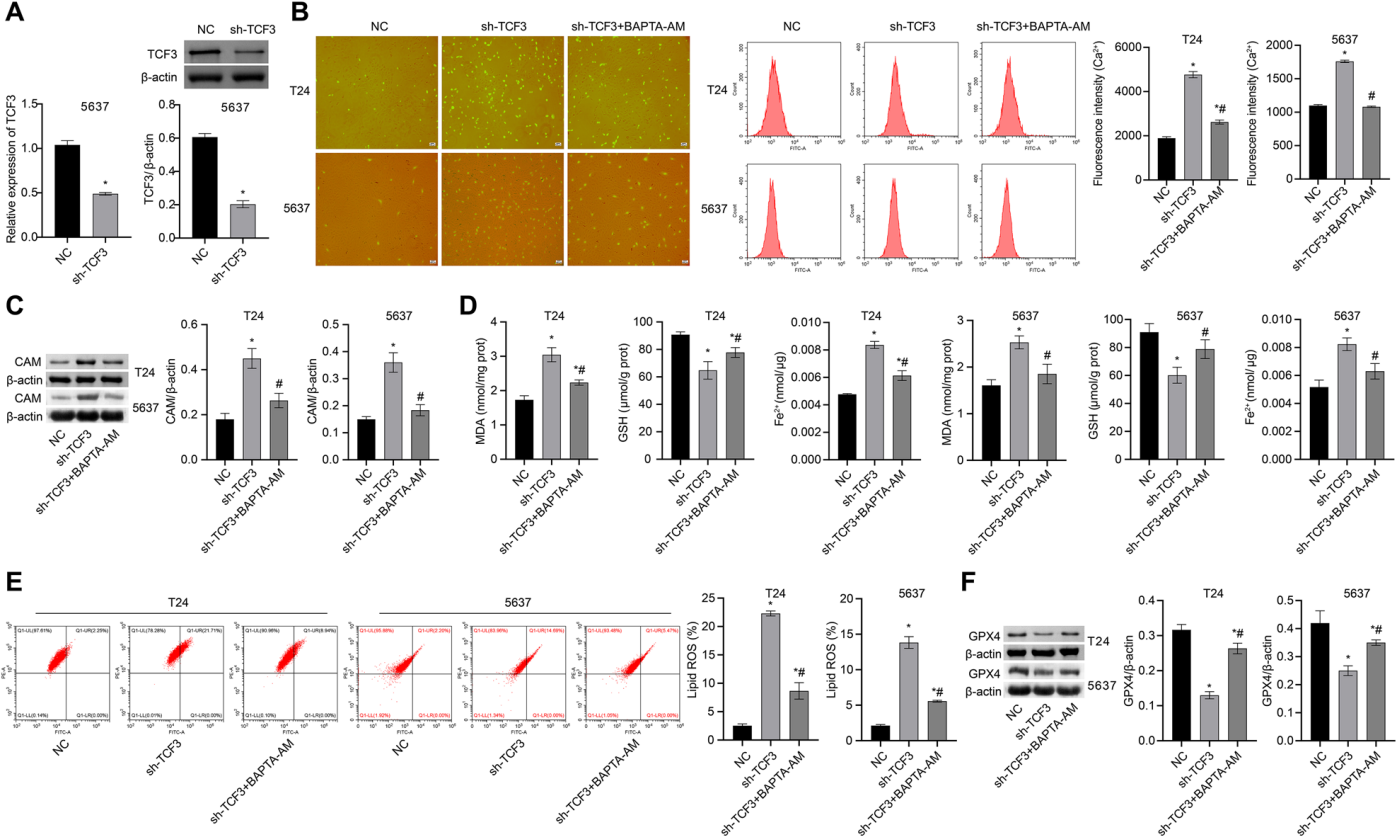
sh-TCF3 stimulates bladder cancer cell ferroptosis through induction of intracellular Ca^2+^ concentration. **A** The expression of TCF3 was measured by qRT-PCR and western blot. **B** Ca^2+^ concentration was measured by Ca^2+^ assay kit (scale bar: 100 μm). **C** CAM expression was obtained by western blot. **D** MDA, GSH and Fe^2+^ levels were measured using biochemical assay kits; **E** Lipid ROS levels were detected by c11-bodipy fluorescent probe; **F** GPX4 expression was obtained by western blot. *p < 0.05, vs. NC; ^#^*p* < 0.05, vs. sh-TCF3.

sh-TCF3 effectively suppressed the expression of TCF3 (refer to Fig. 6A). To further explore the impact of TCF3 on bladder cancer development by influencing intracellular Ca^2+^ concentration and ferroptosis, we treated the cells with BAPTA-AM in combination with sh-TCF3. Following sh-TCF3 treatment, there was a notable increase in Ca^2+^ concentration, CAM expression, as well as MDA, Fe^2+^, and lipid ROS levels, accompanied by a significant decrease in GSH and GPX4 levels. The application of BAPTA-AM reversed these changes observed in the sh-TCF3 group, indicating that sh-TCF3 promoted ferroptosis by elevating intracellular Ca^2+^ concentration (Fig. 6B–F).

### In vivo experiments further demonstrate that TCF3 promotes bladder cancer cell development by inducing Ca^2+^-dependent ferroptosis resistance

**Fig. 7:**
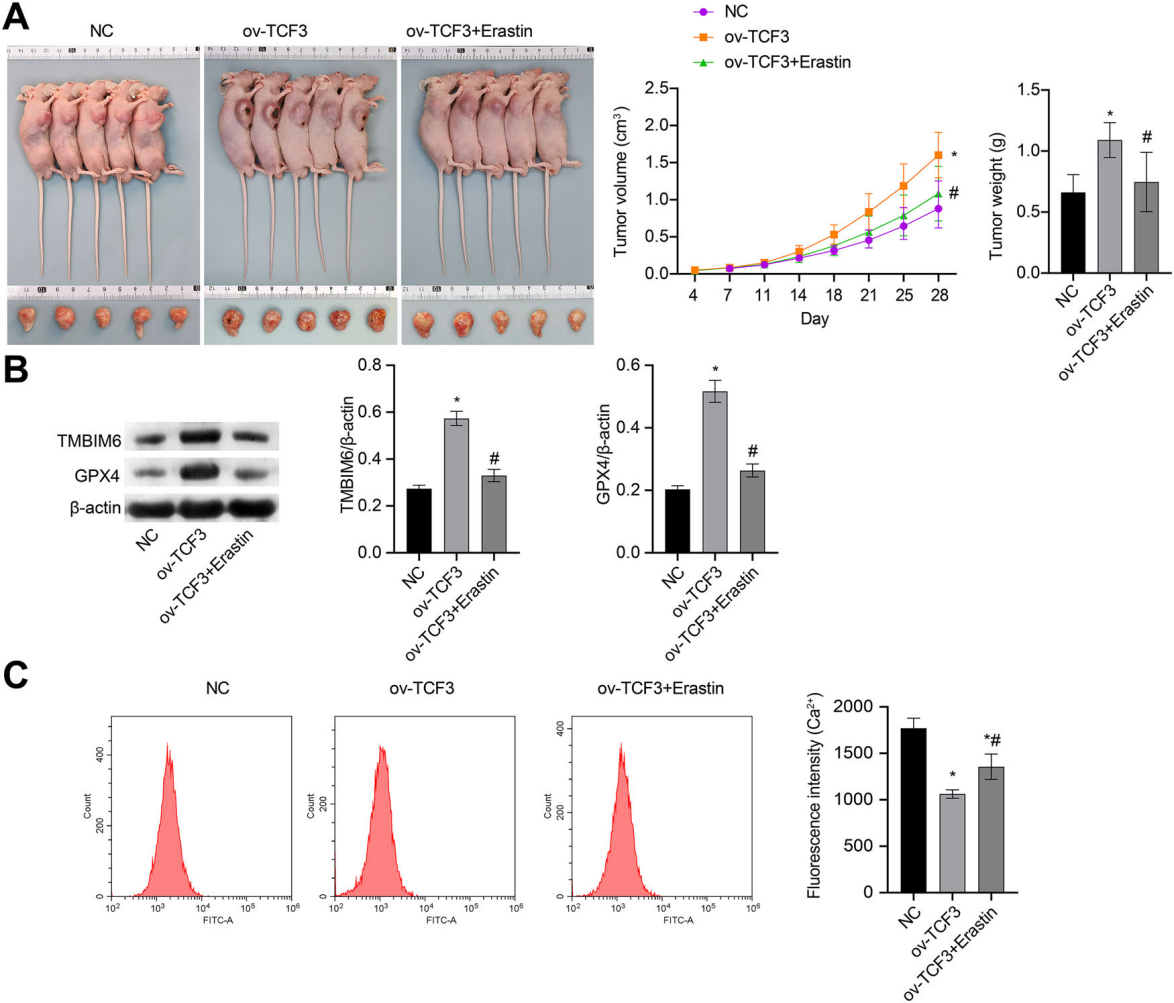
In vivo experiments demonstrate that TCF3 promotes bladder cancer cell development by inducing Ca^2+^-dependent ferroptosis resistance. **A** Representative figures of nude mouse tumors and volume and weight statistics. **B** The TMBIM6 and GPX4 levels were detected by western blot. **C** Ca^2+^ concentration was detected by a Ca^2+^ detection kit (scale bar: 100 μm). *n* = 5; *p < 0.05, vs. NC; ^#^*p* < 0.05, vs. ov-TCF3.

To validate in vitro findings, we conducted animal experiments involving TCF3 overexpression alone and in combination with Erastin treatment. ov-TCF3 led to a notable rise in tumor volume, weight, and levels of TMBIM6 and GPX4, while causing a significant decrease in tumor Ca^2+^ concentration. The application of Erastin reversed these effects, indicating that TCF3 facilitated the progression of bladder cancer cells by promoting resistance to ferroptosis (Fig. 7A–C).

In summary, our research demonstrates that TCF3 promotes the development of bladder cancer by inducing Ca^2+^-dependent ferroptosis resistance through its interaction with TMBIM6.

## Discussion

In this study, we observed elevated expression levels of TMBIM6 and ferroptosis-related proteins (GPX4, SLC7A11, and FTH1) in bladder cancer tissues, while CAM expression was significantly downregulated. Functional experiments demonstrated that siRNA-mediated knockdown of TMBIM6 effectively suppressed bladder cancer cell proliferation through the induction of ferroptosis. Notably, the oncogenic effects induced by TMBIM6 overexpression were significantly attenuated by Erastin treatment. Mechanistically, we identified TCF3 as a transcriptional regulator that directly binds to the TMBIM6 promoter and activates its expression. Furthermore, our findings suggest that TCF3-mediated regulation of TMBIM6 modulates Ca^2+^-dependent ferroptosis, thereby promoting cancer progression. These results were consistently validated through in vivo tumorigenesis assays, providing robust evidence for the functional significance of this molecular pathway in bladder cancer development.

TMBIM6 has been reported to exhibit elevated expression levels in various malignancies, including fibrosarcoma, cervical, breast, lung, nasopharyngeal, and prostate cancers [19, 22]. Within the context of cancer biology, GPX4 serves as a pivotal regulator and central inhibitor of ferroptosis [25], while FTH1 functions as a crucial iron storage protein [26]. Notably, both GPX4 and FTH1 have been shown to be significantly upregulated in clinical specimens from 30 human gastric tumor cases [27]. Furthermore, SLC7A11, which plays an essential role in cystine import for glutathione biosynthesis and antioxidant defense, is frequently overexpressed across multiple human cancers [28, 29]. This overexpression has been particularly implicated in conferring ferroptosis resistance in lung cancer stem-like cells [30]. The calcium signaling pathway, mediated through CaM (the primary Ca^2+^ sensor in eukaryotic cells), exerts extensive regulatory effects on cellular functions. The Ca^2+^/CaM complex, along with Ca^2+^ itself, modulates the activity of numerous enzymes, channels, signaling molecules, and structural proteins [31]. In gastric cancer pathogenesis, elevated expression of CALM2 has been observed in both tissue samples and cell lines [32]. Building upon these previous findings, our study has made a novel observation in bladder cancer: we identified significantly elevated expression levels of TMBIM6 and ferroptosis-related proteins (GPX4, SLC7A11, and FTH1) in clinical bladder cancer specimens. Interestingly, in contrast to these upregulated markers, CAM expression was found to be substantially reduced in bladder cancer tissues.

Erastin, a well-characterized ferroptosis inducer, has been demonstrated to effectively trigger ferroptosis and consequently inhibit tumor progression across various cancer types, including breast, prostate, melanoma, and bladder cancers [33–36]. In the context of bladder cancer specifically, baicalin and Fin56 have been shown to induce ferroptosis through distinct molecular mechanisms, targeting FTH1 and GPX4, respectively [11, 37]. In line with these established findings, our study confirmed that Erastin-mediated induction of ferroptosis significantly suppressed bladder cancer cell progression. While previous research has established that TMBIM6 modulates intracellular Ca^2+^ dynamics to regulate ER stress-induced cell death in various cellular models, including HeLa, HT1080, and MEFs [22, 38], its potential role in ferroptosis regulation, particularly in bladder cancer, remains unexplored. Our investigation revealed a parallel between the effects of TMBIM6 knockdown and Erastin treatment on ferroptosis induction in bladder cancer cells. TMBIM6 may promote tumor development by inducing ferroptosis resistance in bladder cancer cells, which is an innovative point of our study. This discovery not only expands our understanding of TMBIM6’s oncogenic functions but also provides new insights into the molecular mechanisms underlying ferroptosis resistance in bladder cancer.

TCF3, a pivotal transcription factor, serves integral roles in embryonic development, stem cell conservation, and cancer progression. It stands significantly involved in the pathogenesis of various human cancers [39, 40]. TCF3 functions as a tumor suppressor in endometrial cancer [41]. TCF3 enhances cell proliferation and invasion in triple-negative breast cancer by activating transcription [40]. However, TCF3 has been largely unstudied in bladder cancer. Our study found that TCF3 expression was significantly upregulated in bladder cancer cells. In addition, GSEA analysis revealed significant enrichment of TCF3 in several critical biological pathways, including cellular iron ion homeostasis, calcium ion response, calcium ion transmembrane transport, and cellular response to calcium ion signaling pathways. These findings suggest a potential association between TCF3 and both the Ca^2+^ signaling pathway and ferroptosis processes.

Ferroptosis is prevented by MS4A15, which depletes luminal Ca^2+^ stores and reprogrammes membrane phospholipids to be ferroptosis-resistant [42]. MAP30 from Momordica charantia induces an increase in intracellular Ca^2+^ concentration, triggering ROS-mediated ovarian cancer cell death through apoptosis and ferroptosis [43]. The natural product, Erianin, inhibits lung cancer cell growth by inducing Ca^2+^/CAM-dependent ferroptosis [23]. The mechanisms of ferroptosis and Ca^2+^ signaling regulation associated with bladder cancer are more studied [11, 16, 37]. For example, cytokine release in Bacillus Calmette-Guérin-induced bladder cancer cells is regulated by Ca^2+^ signaling [16]. However, Ca^2+^-dependent ferroptosis has not been reported in bladder cancer. Our findings demonstrate that TCF3 directly interacts with TMBIM6 and transcriptionally upregulates its expression, thereby mediating Ca^2+^-dependent ferroptosis resistance and facilitating bladder cancer progression. Our study has not explored the effects of other transcription factors on bladder cancer and Ca^2+^-dependent ferroptosis, which is a limitation of our study.

Our findings demonstrate that TCF3 plays a pivotal role in regulating critical cellular processes, including proliferation, invasion, Ca²⁺ homeostasis, and ferroptosis, highlighting its potential as a novel therapeutic target. The identification of TCF3 as a key regulator in bladder cancer represents a significant advancement in understanding tumor biology and Ca²⁺-dependent ferroptosis-mediated treatment resistance. These insights position TCF3 as a promising candidate for targeted therapy, particularly in bladder cancer, where therapeutic options remain limited.

To translate these findings into clinical applications, our future research will focus on preclinical validation using advanced models, such as patient-derived xenografts and genetically engineered mouse models, to evaluate the therapeutic efficacy of TCF3 modulation in vivo. Furthermore, large-scale clinical cohort studies will be essential to establish the correlation between TCF3 expression levels and patient outcomes, including overall survival, progression-free survival, and treatment response. These efforts will provide a foundation for developing TCF3-targeted therapies and improving prognostic stratification in bladder cancer patients.

In conclusion, our study unveils that TCF3 is significantly overexpressed in bladder cancer, suggesting its role in accelerating bladder cancer advancement by interacting with TMBIM6. This interaction leads to the activation of TMBIM6 expression, thereby enhancing resistance to Ca^2+^-dependent ferroptosis. TCF3 and TMBIM6 emerge as promising biomarkers and therapeutic targets for bladder cancer. The findings of our study provide a basis for the development of targeted therapies focused on Ca^2+^-dependent ferroptosis in bladder cancer.

## Materials and methods

### Human tissues management

Eight pairs of bladder cancer and paracancerous tissues were collected at Huazhong University of Science and Technology Union Shenzhen Hospital. The study protocol was approved by the Huazhong University of Science and Technology Union Shenzhen Hospital Ethics Committee and written informed consent was obtained from all participating patients.

### Cell culture

Five bladder cancer cell lines, including T24 (AW-CCH356), EJ (AW-CCH193), 5637 (AW-CCH141), UM-UC-3 (MEM, AW-CCH041), and BIU-87 (AW-CCH164), along with normal human bladder epithelial cell line SV-HUC-1 (AW-CNH087), were purchased from Abiowell (China). T24, EJ, UM-UC-3, 5637, BIU-87, and SV-HUC-1 cells were cultured in McCoy’s 5A (M9309, Sigma, USA), MEM (M4655, Sigma, USA), RPMI-1640 (R8758, Sigma, USA), and F-12K (N3520, Sigma, USA) mediums respectively. All media were supplemented with 100 U/ml penicillin-streptomycin (10378016, Gibco, USA) and 10% FBS (10099141, Gibco, USA). Cells were cultured in a humid and constant incubator (37 °C, 5% CO_2_, DH-160I, Santn, China).

### Cell treatment

T24 and 5637 cells were transfected with either overexpression plasmids (ov-TMBIM6 or ov-TCF3) or silencing constructs (si-TMBIM6 or sh-TCF3), along with their corresponding negative control (NC) for 48 h. Following transfection, the cells were treated with 5 μM ferroptosis inducer Erastin (#E7781, Selleck Chemicals, USA) or 10 μM Ca^2+^ blocker BAPTA acetoxymethyl ester (BAPTA-AM, B6769, Thermo Fisher Scientific, USA) for 24 h [12, 22]. Ferroptosis inhibitor (Ferrostatin-1, Fer-1, 1 μM, #HY-100579, MCE), apoptosis inhibitor (Z-VAD-FMK, 10 μM, #V116, Sigma, USA), autophagy inhibitor (chloroquine, CQ, 25 μM, C6628, Sigma, USA), or necroptosis inhibitor (necrostain-1, Nec-1, 10 μM, #HY-15760, MCE) were added to 5637 cells for 24 h to assess the effects of TMBIM6 on cell death and autophagy [11]. Certified mycoplasma-free status and STR authentication were confirmed for all cellular models.

### Animals

The required number of experimental animals (15 mice) was calculated based on the degree of freedom in variance analysis and the estimated modeling success rate. A total of 15 male nude mice (4-week-old) were obtained from Hunan SJA Laboratory Animal Co., Ltd. (Changsha, China). The animals were randomly divided into three groups. Each mouse received a subcutaneous injection of 1 × 10^7^ 5637 cells (suspended in 0.1 mL PBS) into the right flank, with cells allocated to three experimental groups: NC, ov-TCF3, and ov-TCF3+Erastin [44]. The experimental period spanned 4 weeks, during which tumor growth was monitored 2 times per week. Tumor progression was systematically observed and recorded. On day 28 post-injection, tumor tissue was removed, weighed, and subsequently processed for further experimental analyses.

### Immunohistochemistry (IHC)

Following baking, the sections were dewaxed to water. Sections were then subjected to antigenic thermal repair. After the inactivation of endogenous enzymes, the sections were incubated overnight at 4 °C with the following primary antibodies, including TMBIM6 (26782- 1-AP, 1:200, Proteintech, USA), calmodulin (CAM, ab45689, 1:200, Abcam, UK), GPX4 (67763-1-Ig, 1:200, Proteintech, USA), SLC7A11 (26864-1-AP, 1:200, Proteintech, USA), FTH1 (ab231253, 1:200, Abcam, UK), and TCF3 (ab224638, 1:100, Abcam, UK). Subsequently, the sections were then incubated with the secondary antibody for 30 min at 37 °C. Sections were stained, then blocked for observation and photographed under a fluorescence microscope (BA410E, Motic, China).

### Quantitative Real-time PCR (qRT-PCR)

Total RNA from the cells was extracted by TRIzol® reagent (15596026, Thermo, USA). The extracted RNA was then reverse transcribed into cDNA by reverse transcription kit (CW2569, CWBIO, China) and subjected to qRT-PCR using a fluorescent quantitative PCR instrument (PIKOREAL96, Thermo, USA). β-actin was used as an endogenous control gene. Three replicate analyses were performed for each sample. Data were evaluated by 2^-ΔΔCT^. The thermal cycling procedure consisted of holding at 95 °C for 10 min, followed by 40 cycles at 95 °C for 15 s, and 60 °C for 30 s. Primer sequences were as follows. TMBIM6, F: 5’ CTCTATTCTGCCCCAGAGCG 3’, R: 5’ TCGAACGATCGAAGCTAGGC 3’; TCF3, F: 5’ ACGAGCGTATGGGCTACCA’, R: 5’ GTTATTGCTTGAGTGATCCGGG 3’; β-actin, F: 5’ ACCCTGAAGTACCCCATCGAG 3’, R: 5’ AGCACAGCCTGGATAGCAAC 3’.

### Cell counting kit 8 (CCK8) assay

The cells were added with 10% CCK8 (NU679, Dojindo, Japan) and then incubated for 4 h (37 °C, 5% CO_2_). A Bio-Tek zymograph (MB-530, Bio-Tek, USA) was utilized to analyze the optical density (OD_450 nm_).

### Colony formation assay

The above treated cells (200/well) were seeded and cultured at 37 °C for 2 weeks. Subsequently, 4% paraformaldehyde (N1012, New Sammy, China) was used to fix the cells for 15 min. Next, cells were stained with crystal violet (G1062, Solarbio, China) for 30 min. After image acquisition, the cells were destained. OD_550 nm_ was assessed using a Bio-Tek enzyme marker.

### Detection of malondialdehyde (MDA), glutathione (GSH), and Fe^2+^ levels

MDA (A003-1-2, Nanjing Jiancheng Biotechnology Co., Ltd, China), GSH (A006-2-1, Nanjing Jiancheng Biotechnology Co., Ltd, China), and Fe^2+^ (ab83366, Abcam, UK) levels were assayed according to the manufacturer’s instructions.

### Lipid ROS

After harvesting, cells or tumor tissues were processed using C11-BODIPY (MX5211-1MG, Shanghai Maokang Biotechnology Co., LTD, China) according to the manufacturer’s protocol. Lipid ROS levels were then measured by flow cytometry (A00-1-1102, Beckman, USA).

### Western blot

The BCA protein assay kit (P0010, Beyotime, China) was used to detect protein concentrations. Extracted proteins were transferred to nitrocellulose membranes after gel electrophoresis. The membranes were blocked with 5% skim milk for 1 h at room temperature and then incubated overnight at 4 °C with the following primary antibodies: anti-GPX4 (1:5000, 67763-1-Ig, Proteintech, USA), anti-CAM (1:1000, #35944, Cell Signaling Technology, USA), anti-TMBIM6 (1:1000, 26782-1-AP, Proteintech, USA), and anti-TCF3 (1:1000, AWA43841, Abiowell, China). Anti-β-actin (66009-1-Ig, 1:5000, Proteintech, USA) was used as an internal loading control. The antibody was incubated with diluted secondary antibody Goat anti-Mouse / Rabbit IgG (AWS0001/AWS0002, 1:5000, Abiowell, China) for 90 min at room temperature. Following incubation with enhanced chemiluminescence (ECL) solution (abiowell, AWB0005, China), protein bands were visualized and analyzed using an ECL imaging system (ChemiScope 6100, CLINX, China). The original Western blot data have been provided in the supplementary material.

### Invasion and migration assay

The upper chamber of a Transwell (3428, Corning, USA) pre-coated with Matrigel basement membrane gel (356231, Corning, USA) was inoculated with cells containing basal medium, while the lower chamber was placed in a complete medium (10% FBS). After 48 h of incubation, cells were stained using crystal violet. OD_550 nm_ was assessed using a Bio-Tek enzyme marker. Wound closure was assessed by measuring the scratch width at 0 h, 24 h, and 48 h, following the previously described method [45].

### Detection of Ca^2+^ concentration

After the cells were collected, the Ca^2+^ concentration of the cells was assayed according to the procedure described in the Ca^2+^ Kit (S1056, Beyotime, China). An inverted fluorescence microscope (XD-202, Jiangnan Instrument, China) and a flow cytometer were used to observe and detect the Ca^2+^ levels of the cells, respectively.

### Bioinformatics analysis

The expression profile of TCF3 was obtained from the cancer genome atlas (TCGA) of bladder cancer. Both gene expression box plot and survival plot of TCF3 were generated. Subsequently, we conducted gene set enrichment analysis (GSEA).

### Chromatin immunoprecipitation (ChIP)-qPCR

As described previously [46], immunoprecipitates were obtained using anti-TCF3 or IgG (#61126 or 30000-0-AP, Proteintech) antibodies at 4 °C overnight. The immunoprecipitated DNA was then quantified using qRT-PCR and the data was normalized to the input data. The final fold enrichment was calculated according to the formula: Fold Enrichment = 2 ^(-ΔΔCt [ChIP/NIS])^.

### Dual-luciferase reporter assay

The transcriptional regulation of TMBIM6 promoter activity by TCF3 was investigated. Briefly, cells were seeded in 96-well plates and co-transfected with psiCHECK-2-TMBIM6, or its corresponding NC, while oe-TCF3, sh-TCF3, or its corresponding NC was used to co-transfect the cells. The dual-luciferase reporter analysis system (E1910, Promega, USA) was applied to assess the relationship between TCF3 and TMBIM6.

### Statistical analysis

The study adhered to randomized and blinded experimental protocols throughout all procedures. Three independent experiments were conducted, and the experimental results were shown as mean ± standard deviation. Statistical analyses were initiated by assessing the normality of distribution patterns using the Shapiro-Wilk test for both normal and log-normal distributions. For normally distributed datasets, intergroup comparisons were conducted using Student’s t-test. When evaluating temporal variations and group differences across multiple time points, a two-way analysis of variance (ANOVA) followed by Bonferroni’s multiple comparison test was systematically applied. For cross-sectional comparisons involving three or more experimental groups, statistical significance was determined through one-way ANOVA with Tukey’s honestly significant difference (HSD) post hoc analysis. *p* < 0.05 was considered to indicate a statistical difference. All statistical analyses were performed using GraphPad 8.0 software.

## Supplementary information

Supplemental Material

## Data Availability

All data analyzed in this study are included in this paper. Further data are available upon reasonable request from the corresponding author.
