# Supplementary figures and images for: Transcription factor TCF3 promotes bladder cancer development via TMBIM6-Ca2+-dependent ferroptosis
Source: Cell Death Discov. 2025 Jul 3;11:303. doi: 10.1038/s41420-025-02585-8 (PMC12229597; doi:10.1038/s41420-025-02585-8)

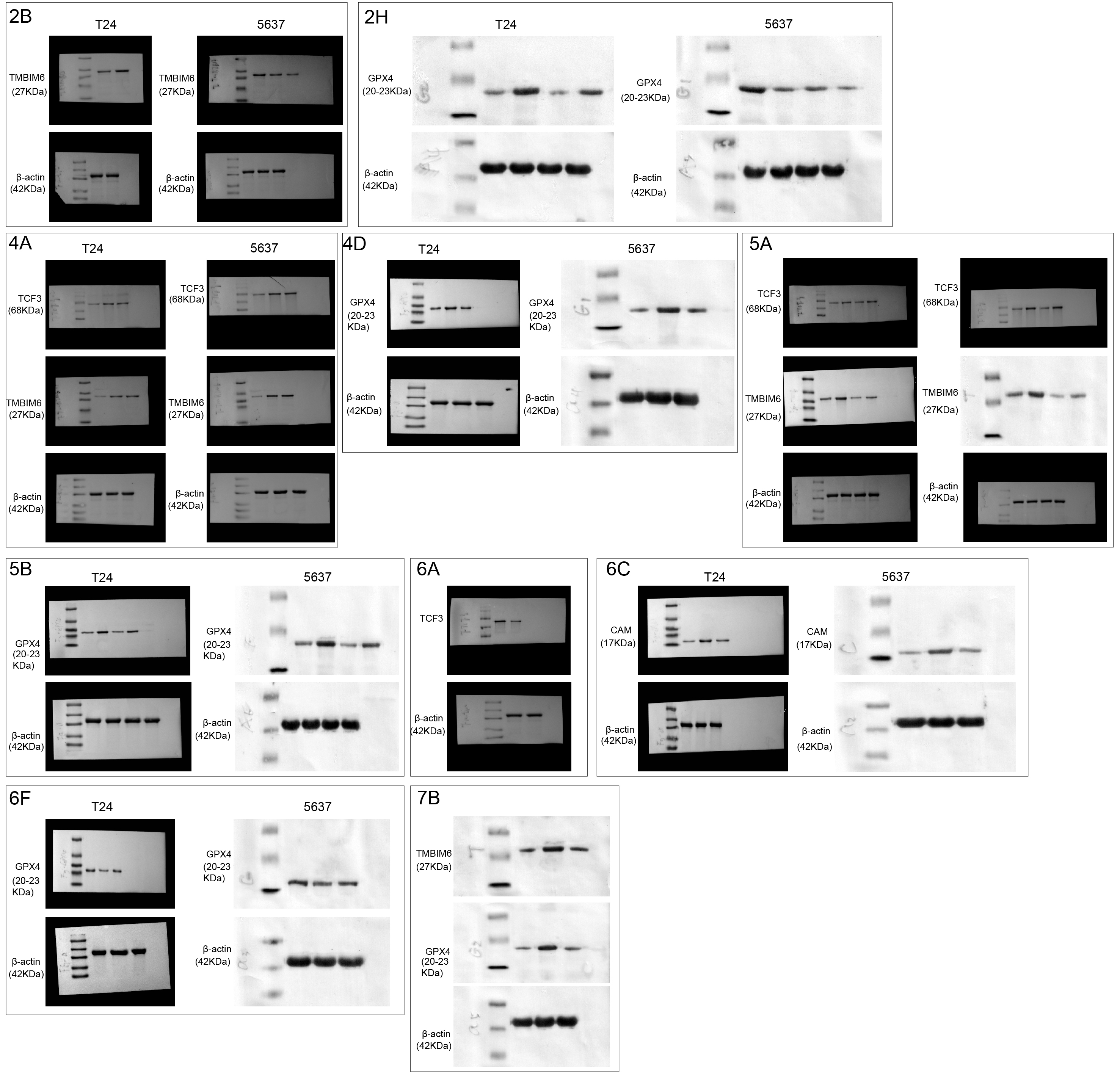

Supplement: Supplementary file 1 — Supplemental Material [file 41420_2025_2585_MOESM1_ESM.jpg]
